# Self-Esteem and Risk Behaviours in Adolescents: A Systematic Review

**DOI:** 10.3390/bs14060432

**Published:** 2024-05-22

**Authors:** Elena Martínez-Casanova, María del Mar Molero-Jurado, María del Carmen Pérez-Fuentes

**Affiliations:** Department of Psychology, University of Almería, 04120 Almería, Spain; mmj130@ual.es (M.d.M.M.-J.); mpf421@ual.es (M.d.C.P.-F.)

**Keywords:** self-esteem, risk behaviours, systematic review, cross-sectional studies

## Abstract

Adolescence is recognised as a notoriously vulnerable period in the human life cycle. Influenced by a complex interplay of biological, psychological and social factors, adolescents show a marked propensity to engage in risk behaviours. A systematic review was conducted of studies published in the Web of Science, PsycInfo and MEDLINE databases over the last decade, with the aim of collecting studies on the relationship between self-esteem and risk behaviour in individuals aged 12–18 years. The aim was to confirm the role of high self-esteem as a consistent protective factor against risk behaviour. The results show that self-esteem is negatively related to risk behaviour. Our results also reflect the need for further research on how sociodemographic factors, among others, affect the relationship between self-esteem and risk behaviours. This review highlights the relevance of implementing specific educational interventions to strengthen self-esteem in adolescents, with the aim of preventing various risk behaviours that may emerge during adolescence and persist throughout life if not addressed early.

## 1. Introduction

Adolescence is a stage of vulnerability to risk behaviours, due to a complex interaction of biological, psychological and social factors, including the brain’s sensitivity to emotions generated by rewarding experiences, the search for identity and the desire for independence, the lack of tools to manage different emotions and, finally, the adolescent’s permeability to the example offered by their primary caregiver [1,2]. These factors can be defined as risk factors, understood as individual attributes and/or characteristics, conditions and/or environmental contexts that increase the likelihood of developing risk behaviours [3].

Risky behaviours encompass any action that threatens biological, psychological or social development [4], with an immediate effect that may be pleasant but with harmful long-term consequences, as they are linked to high morbidity and mortality [5]. This is clearly exemplified in the case of tobacco and alcohol use, which have caused more than 8 million and 3 million deaths, respectively [6,7]. Around 90% of adult smokers admit to having started smoking during adolescence [8], underlining the need for early interventions. In addition, risk behaviours are often associated with each other. For example, drug use is associated with risky sexual relationships [4] or with antisocial behaviour [9]. Another relevant aspect is the connection between eating disorders, which often occur during adolescence [10], and suicide [11].

In order to address risky behaviours, research on protective factors or, in other words, personal or social resources that reduce the tendency to develop risky behaviours and, consequently, health problems, is crucial [3].

Self-esteem, defined as the global evaluation of oneself [12], has traditionally been considered a protective factor against various risk behaviours. It has been linked to a decrease in the consumption of substances such as alcohol, tobacco [13] and illicit drugs, as well as to a lower risk of eating disorders (EA) [14], self-harm, suicidal behaviour [15,16], sexual risk behaviours [17], harmful media use [18,19] and antisocial behaviours such as aggression, delinquency, bullying, cyberbullying and sexting [20,21,22,23,24,25,26]. In addition, some studies report a mediating role of self-esteem, for example, between strict parenting and suicidal ideation [27] or between depression and suicidal ideation [28].

However, recent research has challenged this perspective. Some have found no significant relationship [29,30,31,32] and others have even found a positive relationship [33,34]. In addition, it is important to consider possible variations in the relationship between self-esteem and risk behaviours by gender. Self-reports suggest that women tend to report lower levels of self-esteem compared to men, which may influence how these behaviours manifest themselves in each group [35].

Thus, new research is needed to provide a clear and updated perspective on the subject, in order to determine whether self-esteem is a relevant individual variable for the reduction of risk behaviours in adolescence. The present review aims to find out whether high self-esteem functions as a protective or risk factor by analysing the scientific literature of the last decade, in Spanish and English.

## 2. Materials and Methods

This review has been conducted in accordance with the PRISMA 2020 guidelines for systematic reviews [36].

### 2.1. Data Sources and Search Strategy

The search for publications was conducted between November 2023 and February 2024. Due to the multidisciplinary approach of the review, MEDLINE, Web of Science and PsycInfo databases were consulted. In all three databases, the terms “self-esteem”, “risk behaviours” and “adolescents” were used in English and Spanish. To find documents that included the above terms, the Boolean operators “AND” and “OR” were used. Thus, the search strategy was (autoestima OR self-esteem) AND (conductas de riesgo OR risk behaviors) AND (adolescentes OR adolescents). A total of 7189 citations were identified in the three databases searched. The search was limited to open access journal articles published in English or Spanish between 2013 and 2023, which reduced the number of citations to 1984. Table 1 shows the search strategy and the terms included.

### 2.2. Elegibility Criteria

Articles were included if they (1) had adolescent participants (12–18 years); (2) were quantitative and cross-sectional; (3) were in English or Spanish; (4) treated self-esteem as a unidimensional concept; (5) and addressed a specific risk behaviour.

Articles were excluded if they (1) did not provide clear data on the age of participants or if ages were below 12 years or above 18 years; (2) had gender bias in the sample; (3) included participants with physical, psychological or social particularities; (4) considered terms such as “self-concept” or “self-image” as synonymous with self-esteem; (5) superficially addressed risk behaviours; (6) focused on the creation, validation or adaptation of scales and questionnaires; (7) evaluated the effectiveness of intervention programmes; (8) and/or were limited in their accessibility.

### 2.3. Quality of Manuscripts

Two reviewers independently rated the quality of the selected full-text articles using the Newcastle–Ottawa Scale (NOS) tool adapted for cross-sectional studies by Herzog et al. [37]. A minimum score of 5 out of 10 was set for this review to be selected.

### 2.4. Data Extraction

**Figure 1:**
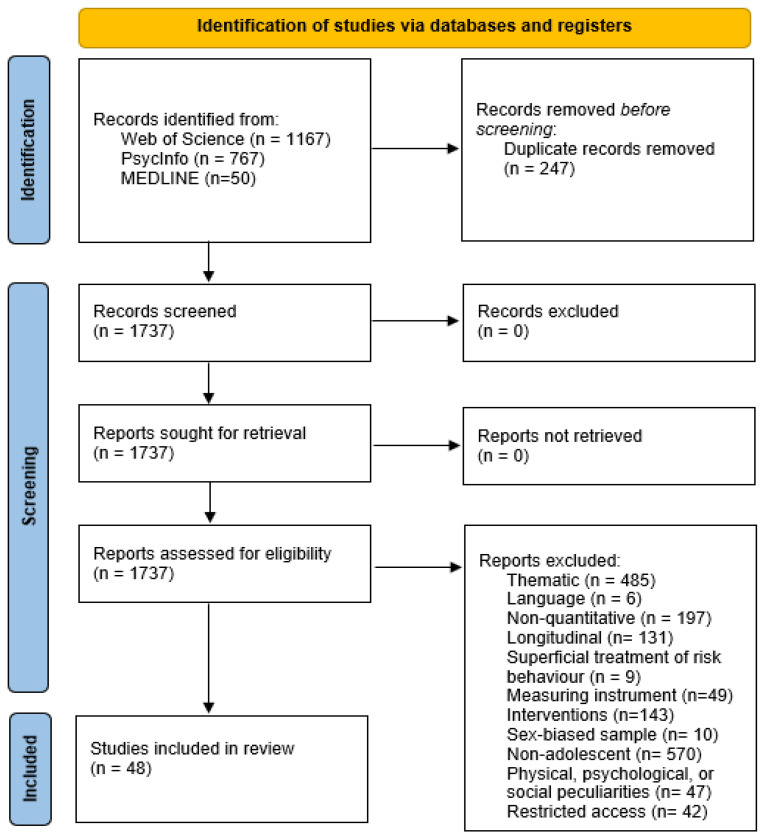
Flowchart of the document selection process.

Studies were selected following a two-stage process, as shown in Figure 1 (PRISMA flowchart): (1) screening of the titles and abstracts of potentially eligible studies followed by (2) screening of the full texts of the preliminarily selected articles. The assessment of each record was performed by two reviewers.

A total of 1737 articles were identified as eligible; however, after full-text analysis, only 48 articles remained and were included in this review.

## 3. Results

Self-esteem’s impact on identity and behaviour is widely studied. This systematic review examines the available evidence on the interaction between self-esteem and risk behaviours in adolescents, providing a comprehensive overview of how self-perception may influence participation in activities that carry potential negative consequences for health and well-being. The salient features of the studies included in the current review are summarised in Table 2.

Six main categories of risk behaviours have been identified: (1) substance abuse, (2) eating disorders, (3) suicide and self-harm, (4) risky sexual practices, (5) harmful use of media and (6) antisocial behaviour. According to our inclusion and exclusion criteria, the most researched category was “suicide and self-harm”, while the least studied categories were “risky sexual practices” and “antisocial behaviour”.

In terms of the year of publication, 2022 stands out as the year with the highest number of publications, coinciding with the return to normality after the COVID-19 pandemic. It is notable that in 2021, during the height of the pandemic, research experienced a significant decrease. There is a notable increase in the number of publications from 2021 to 2022, followed by a similar transition between 2016 and 2017.

With regard to the country of publication, there is evidence of international interest in the subject. However, Spain stands out as the country with the highest number of publications related to self-esteem and risk behaviours.

As for the sample sizes of the studies analysed, there is considerable disparity, ranging from a minimum of 100 to a maximum of 57,767 subjects. As for the age of the subjects, they ranged from 12 to 18 years of age, with 15 and 16 years of age standing out, present in more than 40 of the 48 studies included.

Regarding the results, on the one hand, in those obtained in the category of substance abuse, a negative relationship prevails with alcohol consumption [40,41,45,46], although one study highlights that this relationship is only significant in adolescents of high socioeconomic status [44]. With regard to tobacco, the negative relationship also predominates [42,45], although one study notes that this relationship is significant only with cigarettes and not with hookah smoking [39] and another reports a non-significant relationship [40]. For cannabis, two studies report no or a weak relationship [38,40], respectively, and two others report a relationship [43,45], especially in older men [43], requiring further research to provide clarity on the relationship between these two variables.

On the other hand, in the category of eating disorders, all studies recognise a negative relationship between eating disorders and self-esteem [47,48,49,50,51,52,53,54,55]. Furthermore, a relevant aspect of the role of self-esteem is highlighted in the study by Cella et al. [53] where self-esteem is identified as a mediator in the relationship between parental bonding and the incidence of binge eating, providing a more complete understanding of how self-esteem is related to eating disorders.

Regarding the category of suicide and self-harm, all studies report a negative relationship between self-esteem and self-harm, both suicidal and non-suicidal [56,57,58,59,60,61,63,64,65,66,67]. Furthermore, Brausch and Decker’s [62] study broadens the understanding of how self-esteem affects suicide by identifying a modulatory effect, revealing that the relationship between depression and suicidal ideation is mainly manifested in individuals with low levels of self-esteem.

With regard to sexual risk behaviour, the evidence shows that there is a predominantly negative relationship between self-esteem and sexual risk behaviour [68,69,72]. One study partially agrees with these results, maintaining a negative relationship in women, while in men, the relationship is positive [70], and another disagrees completely, finding no significant relationship [71].

In terms of harmful media use, evidence shows a negative relationship between self-esteem and problematic information and communication technology (ICT) use [73,74,76,77,80], although one study partially agrees with the results, finding no significant relationships in men [75], and another finds that this relationship is mediated by depression and that the influence of depression on the relationship between self-esteem and problematic ICT use is moderated by interpersonal trust [78]. Wang’s [79] study points to a mediating role between parent–child closeness, peer closeness and problematic Internet use.

And finally, with regard to antisocial behaviour, the evidence collected points to a negative relationship between self-esteem and antisocial behaviour, particularly in cases of violence [82,83,84], in contrast to those studies that find no significant relationships [85]. Regarding delinquency, Gauthier-Duchesne et al. [81] recognise the mediating role of self-esteem between the experience of sexual abuse and delinquency, as well as highlighting the fact that it is men who have lower self-esteem and higher delinquent behaviour. However, among those who have not experienced sexual abuse, boys typically display higher self-esteem than girls. Interestingly, the data show that higher self-esteem correlates with increased delinquent behaviour.

### Quality of the Manuscripts

All articles used in this review scored well (Table 3) and were therefore considered to be of good quality.

## 4. Discussion

The aim of this study was to examine the relationship between self-esteem and risk behaviour in adolescents and to determine whether self-esteem, high or low, was a consistent protective factor against risk behaviour. In exploring these dynamics, our results also uncovered significant relationships between various risk behaviours, such as the association between depression and suicidal ideation [62] or between substance use and problematic Internet use [38], which not only bolsters the findings of prior research [4,9,11] but also underscores the necessity and significance of investigating protective factors in contrast to risk behaviours [3].

In regard to self-esteem as a protective factor, our results show a predominant and negative relationship between self-esteem and all the risk behaviours analysed, including tobacco and alcohol use, eating disorders, non-suicidal self-harm and suicide, sexual risk behaviours, problematic ICT use and antisocial behaviour, and these results are supported by the previous literature [14,15,17,18,20,21,22,24], with the exception of cannabis use, where our findings are ambiguous due to us finding equality between studies supporting a negative relationship and those finding no significant associations. There is no clear trend of a negative relationship, as seen with the rest of the analysed risk behaviours, nor a predominant relationship, even if not negative. The previous literature has already identified this scenario, with some studies indicating a negative relationship between cannabis use and self-esteem [14], while others found no significant relationship [29]. In this context, our findings highlight the need for further research to clarify the relationship between cannabis use and self-esteem. These results can be better understood by examining the existing literature, which has traditionally focused more on substances like alcohol or tobacco [6,7,8]. This historical preference for certain substances may have limited the amount of research focused on cannabis and, therefore, could explain the scarcity of studies dedicated to understanding its effects and associated risks.

Furthermore, our study provides further insight into the mechanisms underlying this relationship by recording, on the one hand, the mediating role of self-esteem between parental bonding and the incidence of binge eating [53], between the experience of sexual abuse and involvement in delinquent activities [81], between adolescents’ closeness to parents and friends and problematic Internet use [79] and, on the other hand, by recording the modulating role of self-esteem in the relationship between depression and suicidal ideation [62]. These results suggest that self-esteem is not only an important factor in its own right but may also influence how other experiences and factors affect adolescents’ behaviour. The previous literature also reported a mediating role of self-esteem, for example, between strict parenting and suicidal ideation [27] or between depression and suicidal ideation [29].

This implies that high self-esteem is a protective factor and low self-esteem acts as a risk factor. However, it is important to note that these significant relationships may vary according to several factors, such as gender [70,75] or socioeconomic status [44]. With regard to gender, our results show that women report lower self-esteem [75], in line with the previous literature [35], and therefore, taking as a reference the results of our study that indicate a prevalent negative relationship between self-esteem and risk behaviours, they are more vulnerable to engaging in risk behaviours [58], aligning with the prior literature [14]. The few studies reporting that males are more vulnerable usually coincide with the minority of studies finding a positive relationship between self-esteem and risky behaviour [81].

Although our findings reflect a higher incidence of studies showing a negative relationship between self-esteem and risk behaviours, there is a minority of studies suggesting a positive relationship. These differences are more fully explained when considering a multidimensional view of self-esteem, in line with the proposal of Salmivalli et al. [35], who show that it is necessary to go beyond high or low self-esteem and take into account the “dark side” of self-esteem that the traditional literature, with a unidimensional view of self-esteem, has ignored.

In addition, the vulnerability to risk behaviours may be influenced by a combination of individual, social, economic and environmental factors that interact in complex ways [1,2], suggesting a multifaceted picture that requires the adoption of an ecological approach, as referenced by Bronfenbrenner, for comprehensive understanding.

Societal pressures and expectations can exert a significant influence on how adolescents perceive their self-esteem. However, it is important to recognise that not all cultures adhere to the same principles. For instance, in Slovenia, the normalisation of alcohol consumption illustrates how cultural norms can vary widely. In this context, socialising and gathering often involve alcohol, contributing to its normalisation as a means of social interaction. In such a cultural milieu, individuals who do not partake in drinking may perceive their self-esteem diminishing, as they may feel pressure to conform to group norms in order to feel accepted and valued. These relationships are stronger among the female sex, as the female sex is primarily driven by situational and socio-psychological factors, while the male sex is determined to a greater extent by their personality [35,44]. This dynamic highlights how cultural context can shape individuals’ behaviours and perceptions of self-worth. However, it is worth noting that personal characteristics also play a role in how individuals respond to societal pressures. Some individuals may resist the influence of cultural norms and maintain their self-esteem despite external pressures to conform [1,2,41,46].

Interestingly, in Slovenia, individuals from higher socioeconomic backgrounds may not experience a decline in self-esteem if they choose not to drink. This could be attributed to different expectations and standards held by individuals in these social circles. In such contexts, individuals may place greater emphasis on personal achievements and values beyond social conformity, thereby maintaining their self-esteem independent of cultural norms surrounding alcohol consumption. Although not everything is explained by self-esteem and it is necessary to look at each situation in an ecological way, self-esteem plays a fundamental role in adhering, to a greater or lesser extent, to these prevailing values of the moment, since in this process, self-esteem can both affect and be affected by the social role that is assumed, generating a continuous cycle of mutual influence between both aspects [1,2,35,44].

Consequently, the question arises whether problematic and risky behaviours are a reflection of a society with self-esteem problems, which adheres to prevailing social values out of an uncontrollable need to belong. Today, tackling this endless cycle and fostering self-esteem represents a considerable challenge, mainly due to the powerful influence of social media and influencers on adolescents [83]. These actors have become major socialisation agents, often challenging the authority of other socialisation agents. It is crucial to examine how these contextual and socio-demographic variables interact with self-esteem to influence the propensity for risky behaviours. This becomes imperative in order to develop more accurate and effective prevention and intervention strategies.

## 5. Conclusions

This study provides a compilation of diverse research that examines the relationship between self-esteem and various risk behaviours. This compilation has allowed us to fulfil our main objective: to conduct a comprehensive analysis detailing the connection between levels of self-esteem and risk behaviours.

However, this study has some limitations, including the restriction imposed by our exclusion criterion, which left out those articles in which the sample had physical, psychological or social peculiarities. Although we implemented this criterion with the intention of ensuring the representativeness of the results, paradoxically, there is a risk of excluding certain groups and affecting the generalisability of the findings. Furthermore, by limiting our acceptance to exclusively quantitative studies, we prioritised the collection and analysis of numerical and statistical data. Although these studies provide valuable and objective information, they sometimes lack more descriptive or narrative contexts that could enrich the understanding of the results. Also, by approaching self-esteem as a unidimensional concept, there is a risk of oversimplifying the understanding of this psychological phenomenon, which may limit the validity and applicability of the study results. It would be beneficial to consider including multidimensional measures of self-esteem in order to obtain a more complete and accurate understanding of how this construct relates to risk behaviours. Additionally, it is worth noting that all papers reviewed were open access, which may introduce a bias in the selection of the literature, excluding studies that were not freely available. Finally, the thematic breadth addressed in this study, and, consequently, the large sample collected, has presented a significant limitation in the analysis of the results. The abundance of information has possibly limited the depth of our conclusions and the identification of significant patterns by making it difficult to analyse each subgroup or specific aspect in detail. It is essential to keep these limitations in mind when interpreting the results and to consider possible areas for improvement in future research.

Despite the aforementioned limitations, this study achieves its main objective by providing an update on the relationship between self-esteem and risk behaviours, identifying a general trend that recognises high self-esteem as a protective factor against these behaviours, highlighting the importance of developing interventions or programmes aimed at strengthening adolescents’ self-esteem as a preventive strategy to reduce risk behaviours. Furthermore, our results underline the need to explore factors that may affect this relationship, such as gender and socioeconomic background, in order to design effective interventions, presenting these factors as another solid basis for future research.

## Figures and Tables

**Table 1:** Results obtained in each database.

Database	Language	Search Strategy	No. of Initial Results	No. of Results after Applying Filters
Web of Science	Spanish English	(autoestima OR self-esteem) AND (conductas de riesgo OR risk behaviors) AND (adolescentes OR adolescents)	4542	1167
PsycInfo	Spanish English	(autoestima OR self-esteem) AND (conductas de riesgo OR risk behaviors) AND (adolescentes OR adolescents)	2217	767
MEDLINE	Spanish English	(autoestima OR self-esteem) AND (conductas de riesgo OR risk behaviors) AND (adolescentes OR adolescents)	429	50
Total			7189	1984

**Table 2:** Characteristics of the studies included.

Risk Behaviour	Author	Sample	Country	Objectives. Analyse the Relationship between Self-Esteem and …	Measuring Instrument	Results
Drug abuse	Rial et al. [38]	3882 (12–18)	Spain	cannabis use and self-esteem	RSES	Cannabis⤄SE
	Anbarlouei et al. [39]	1282 (14–17)	Iran	smoking (cigarettes and hookahs)	RSES	Tobacco⟺SE (−) Hookahs⤄SE
	Jongenelis et al. [40]	1661 (15–17)	Australia	expenditure on alcohol, tobacco and cannabis	ASQ	Alcohol⟺SE (−) Tobacco and cannabis⤄SE
	Pérez-Fuentes et al. [41]	1287 (14–18)	Spain	perceived pressure to drink alcohol	RSES	Pressure to drink⟺SE (−)
	Baheiraei et al. [42]	870 (15–18)	Iran	tobacco and alcohol expenditure	CTC-YS	Tobacco⟺SE (−)
	Chen et al. [43]	57,767 (13–16)	United States	marijuana use by age and sex	Agreement Statement	Marijuana⟺SE (−)
	Mehanović et al. [44]	2636 (12–14)	Slovenia	alcohol consumption	EU-Dap questionnaire	Alcohol⟺SE (−) in⬆socioeconomic status
	Ninkron et al. [45]	624 (12–18)	Thailand	substance abuse	Validated instrument	Substance abuse⟺SE (−)
	Pérez-Fuentes et al. [46]	1287 (14–18)	Spain	resistance to pressure to drink alcohol	RSES	Anxiety and expectatives ⟺SE⟺Pressure to drink (−)
Eating disorders	Gutiérrez et al. [47]	448 (12–15)	Spain	eating disorders	RSES	Eating disorders⟺SE (−)
	Ying et al. [48]	356 (13–16)	Malaysia	binge eating	RSES	Binge eating⟺SE (−)
	Gomez-Sanchez et al. [49]	100 (13–16)	Peru	anorexia and bulimia	FRAB	Anorexia and bulimia⟺SE (−)
	Zhao et al. [50]	593 (13–17)	China	food addiction	RSES	Food addiction⟺SE (−)
	Mora et al. [51]	579 (12–16)	Spain	risk of eating disorders	RSES	Eating disorders⟺SE (−)
	Pamies-Aubalat et al. [52]	1630 (12–18)	Spain	eating disorders	RSES	Eating disorders⟺SE (−)
	Cella et al. [53]	973 (12–16)	Italy	binge eating	RSES	Parental bonding ⟺SE⟺Binge eating (−)
	Frieiro et al. [54]	721 (12–18)	Spain	diet, bulimia, preoccupation with food and oral control	RSES	Eating disorders⟺SE (−)
	Olsen et al. [55]	2509 (16)	Denmark	binge eating disorder	SISES	Binge eating⟺SE (−)
Suicide and self-harm	Lee et al. [56]	2258 (15–18)	Republic of Korea	suicidal behaviour	RSES	Suicidal behaviour⟺SE (−)
	Huang et al. [57]	5879 (15–17)	China	self-harm and attempted suicide	RSES	Self-harm and attempted suicide⟺SE (−)
	Muñetón et al. [58]	617 (14–18)	Colombia	suicidal risk	Suicide Orientation Inventory ISO-30	Suicidal risk⟺SE (−)
	Tang et al. [59]	1060 (14–16)	Taiwan	non-suicidal self-harm	RSES	Self-harm⟺SE (−)
	Delfabbro et al. [60]	2552 (14–16)	Australia	suicidal tendencies	RSES	Suicidal tendencies⟺SE (−)
	Fonseca-Pedrero et al. [61]	1790 (14–18)	Spain	suicidal behaviour	RSES	Suicidal behaviour⟺SE (−)
	Brausch and Decker [62]	392 (14–16)	United States	suicidal ideation	RSES	SE⬆: Depression⤄Suicide Ideation
	Oktan [63]	263 (15–18)	Turkey	self-harm and body image	RSES	Self-harm⟺SE (−)
	Garbus et al. [64]	4013 (12–15)	Mexico	suicidal behaviour	RSES	Suicidal behaviour⟺SE (−)
	Vawda [65]	222 (13–15)	South Africa	suicidal behaviour	RSES	Suicidal behaviour⟺SE (−)
	Ybarra et al. [66]	5542 (13–18)	United States	bullying and suicidal ideation	Covariate questionnaire	Suicidal ideation⟺SE (−)
	Lin et al. [67]	2170 (15–16)	Taiwan	non-suicidal self-harm	RSES	Self-harm⟺SE (−)
Risky sexual practices	Kerpelman et al. [68]	680 (15–18)	United States	sexual risk behaviours	RSES	Sexual risk behaviours⟺SE (−)
	Li et al. [69]	771 (14–18)	China	problematic use of pornography on the Internet	RSES	Sexual risk behaviours⟺SE (−)
	Ramiro et al. [70]	1005 (14–18)	Spain	sexual risk behaviours	RSES	Sexual risk behaviours⟺SE (−) in women and (+) in men
	Ssewanyana et al. [71]	296 (12–17)	Kenya	sexual risk behaviour	RSES	Sexual risk behaviours⤄SE
	Thurston et al. [72]	822 (16–18)	South Africa	sexual risk behaviour	RSES	Sexual risk behaviours⟺SE (−)
Harmful use of media	Munno et al. [73]	191 (14–18)	Italy	pathological use of the Internet	MMPI-A	Pathological use of the Internet⟺SE (−)
	Warburton et al. [74]	866 (12–17)	Australia	Internet gaming disruption	RSES	Internet gaming disruption⟺SE (−)
	Barthorpe et al. [75]	4032 (13–15)	United Kingdom	time in social networks	RSES	Time in networks⟺SE (−) in women and ⤄ in men
	Akbari et al. [76]	3375 (13–18)	Iran	problematic use of social media	RSES	Problematic use of social media⟺SE (−)
	Lin et al. [77]	2170 (15–16)	Taiwan	Internet addiction	RSES	Internet addiction⟺SE (−)
	Li et al. [78]	637 (14–17)	China	problematic smartphone use	RSES	SE⟺Depression⟺Use (⬆interpersonal trust=⤄)
	Wang [79]	960 (13–16)	China	problematic Internet use	CSES	Parental bonding ⟺SE⟺Internet use (−)
	Tural and Yeşilova [80]	1150 (15–18)	Turkey	Internet addiction	CSEI	Internet addiction⟺SE (−)
Antisocial behaviour	Gauthier-Duchesne et al. [81]	8194 (14–18)	Canada	crime by gender	Self-description questionnaire	Sexual experience abuse⟺SE⟺Crime (−) Not sexual experience abuse: SE⟺Crime (+)
	Dosil et al. [82]	268 (12–17)	Spain	dating violence	BASC-S3 CDS	Dating violence⟺SE (−)
	Rial et al. [83]	3772 (12–17)	Spain	problematic Internet use	RSES	Problematic Internet use⟺SE(−)
	Garaigordobil [84]	3026 (12–18)	Spain	being a cyber aggressor	RSES	Cyber aggressor⟺SE (−)
	Rębisz et al. [85]	541 (14–15)	Poland	cyberbullying	Scales of the Battery of Social Functioning questionnaires	Cyberbullying⤄SE

SE: self-esteem. RSES: Rosenberg Self-Esteem Scale; ASQ: Adolescent Self-Esteem Questionnaire; SISES: Single-Item Self-Esteem Scale; MMPI-A: Minnesota Multiphasic Personality Inventory-Adolescent; BASC-S3: Behavior Assessment System for Children and Adolescents; CDS: Children’s Depression Scale; CTC-YS: Communities of Concern Youth; FRAB: Risk Factors Scale for Anorexia and Bulimia; CSES: Rosenberg Children’s Self-Esteem Scale; CSEI: Coopersmith SE Inventory. QS: quality score. ⟺ (variables are significantly related); ⤄ (the relationship between the variables is weak or not significant); ⬆ (high level).

**Table 3:** Results of the assessment of the methodological quality of the included studies.

Author	Total Score	Selection				Comparability	Result	
		Representativeness of Sample	Sample Size	Non-Respondent	Exposure Check	Design and Analysis	Evaluation Results	Statistical Test
Rial et al. [38]	7	*	*		**	*	*	*
Anbarlouei et al. [39]	8	*	*	*	**	*	*	*
Jongenelis et al. [40]	6	*	*		**		*	*
Pérez-Fuentes et al. [41]	6	*	*		**		*	*
Baheiraei et al. [42]	7	*	*		**	*	*	*
Chen et al. [43]	7	*	*	*	**		*	*
Mehanović et al. [44]	6	*	*		**		*	*
Ninkron et al. [45]	6	*	*		**		*	*
Pérez-Fuentes et al. [46]	6	*	*		**		*	*
Gutiérrez et al. [47]	6	*	*		**		*	*
Ying et al. [48]	7	*	*	*	**		*	*
Gomez-Sanchez et al. [49]	7	*	*		**	*	*	*
Zhao et al. [50]	7	*	*	*	**		*	*
Mora et al. [51]	6	*	*		**		*	*
Pamies-Aubalat et al. [52]	6	*	*		**		*	*
Cella et al. [53]	6	*	*		**		*	*
Frieiro et al. [54]	6	*	*		**		*	*
Olsen et al. [55]	7	*	*		**	*	*	*
Lee et al. [56]	6	*	*		**		*	*
Huang et al. [57]	8	*	*	*	**	*	*	*
Muñetón et al. [58]	8	*	*	*	**	*	*	*
Tang et al. [59]	8	*	*	*	**	*	*	*
Delfabbro et al. [60]	6	*	*		**		*	*
Fonseca-Pedrero et al. [61]	6	*	*		**		*	*
Brausch and Decker [62]	7	*	*		**	*	*	*
Oktan [63]	6	*	*		**		*	*
Garbus et al. [64]	7	*	*		**	*	*	*
Vawda [65]	6	*	*		**		*	*
Ybarra et al. [66]	6	*	*		**		*	*
Lin et al. [67]	8	*	*	*	**	*	*	*
Kerpelman et al. [68]	7	*	*		**	*	*	*
Li et al. [69]	8	*	*	*	**	*	*	*
Ramiro et al. [70]	6	*	*		**		*	*
Ssewanyana et al. [71]	7	*	*		**	*	*	*
Thurston et al. [72]	6	*	*		**		*	*
Munno et al. [73]	6	*	*		**		*	*
Warburton et al. [74]	7	*	*		**	*	*	*
Barthorpe et al. [75]	7	*	*		**	*	*	*
Akbari et al. [76]	6	*	*		**		*	*
Lin et al. [77]	7	*	*	*	**		*	*
Li et al. [78]	8	*	*	*	**	*	*	*
Wang [79]	7	*	*	*	**		*	*
Tural and Yeşilova [80]	7	*	*		**	*	*	*
Gauthier-Duchesne et al. [81]	6	*	*		**		*	*
Dosil et al. [82]	6	*	*		**		*	*
Rial et al. [83]	7	*	*		**	*	*	*
Garaigordobil [84]	6	*	*		**		*	*
Rebisz et al. [85]	7	*	*	*	**		*	*

SELECTION, maximum 5 asterisks. Representativeness of Sample: * random or non-random sampling. Sample Size: * justified. Non-Respondents: * comparability between respondent and non-respondent characteristics is established and response rate is satisfactory. Exposure Check: ** validated measurement tool; * measurement tool not validated but described or available. COMPARABILITY, maximum 2 asterisks. Design and Analysis: *study controls for the most important confounding factor; * study controls for any additional factors. OUTCOME, maximum 3 asterisks. Evaluation Results: ** independent blinded assessment; ** record linkage; * self-reporting. Statistical Test: * the test used to analyse the data is described and appropriate, and the measure of association, including confidence intervals and probability level (*p*-value), is presented.

## Data Availability

The data presented in this study are available on request from the corresponding author.
